# The Aged Lower Urinary Tract: Deficits in Neural Control Mechanisms

**DOI:** 10.3389/fragi.2021.791833

**Published:** 2021-12-20

**Authors:** Cara C. Hardy

**Affiliations:** ^1^ UConn Center on Aging, UConn Health, Farmington, CT, United States; ^2^ Department of Surgery, University of Connecticut SOM, UConn Health, Farmington, CT, United States; ^3^ CT Institute for the Brain and Cognitive Sciences, University of Connecticut, Storrs, CT, United States

**Keywords:** bladder, cystometry, aging, micturition, neural control

## Abstract

Bothersome urinary symptoms plague many older adults and disproportionally affect women. Underreporting of symptoms and general stigma/embarrassment associated with incontinence has negatively impacted the availability of treatments, as research cannot be championed if the severity of the problem is not apparent. Available therapeutics have limited efficacy and are often not recommended in aged patients. Lower urinary tract function has a long and rich history in animal studies; while much of the underlying anatomy has been described, including neural control mechanisms, the impact of aging has only just begun to be addressed. Recent work has provided strong evidence that neural control over micturition is significantly impacted by aging processes. This mini review discusses recent findings regarding how aging impacts the neural control mechanisms of micturition.

## Introduction

With increasing age comes an increased incidence of lower urinary tract symptoms (LUTS), most prevalent in women and institutionalized populations (Centers for Medicare and Medicaid Services, 2014). The severity in which quality of life suffers with LUTS cannot be stressed enough: a study in hospitalized patients with serious illnesses found that incontinence was most highly considered as a fate worse than death over morbidities such as breathing/feeding tube dependence, dementia, and constant pain (Rubin et al., 2016). Current therapeutics of urologic dysfunction include anticholinergics, phosphodiesterase inhibitors, β3-adrenergic agonists, and nerve blocks such as botulinum toxin (Parker and Griebling, 2015; Andersson, 2016). While therapeutic interventions are available, current treatments are limited in their efficacy with many undesirable side effects (Centers for Medicare and Medicaid Services, 2014). Promising new treatments have been aimed at modulating afferent activity (Hood and Andersson, 2013; Andersson, 2016).

The bladder demonstrates complex neural regulation of its function despite a seemingly simple design. Control over the urinary bladder occurs as both an involuntary reflex and a conscious process. In cortical regions of the central nervous system (CNS), cortical function acts to suppress the voiding reflex until micturition is desired (de Groat et al., 2015). Conversely, this cortical influence can voluntarily initiate voiding at subthreshold volume/pressure. With maturation, control over this delicate system is refined: toddlers undergoing potty training are training not only their bladders but also the brain's perception and sensitivity to bladder sensations (Eastham and Gillespie, 2013). The association of sensations/perceptions of bladder volume with intentional inhibition of the voiding reflex is a learned behavior that strengthens as the CNS continues to develop. With aging and diseases of aging such as neurodegeneration, the precision of control over micturition achieved during maturation/development is lost and results in LUTS.

## Neural Control of Micturition

CNS control of the lower urinary tract works *via* both somatic (conscious) and autonomic (unconscious) pathways and has been previously well reviewed (see Fowler and de Groat, 2008) (Fowler et al., 2008). The autonomic voiding reflex is triggered by a *threshold pressure*, the point at which the bladder switches from a state of urine storage to urination. Information regarding bladder content is relayed through afferent fibers to the CNS. Afferent activity is conveyed to the periaqueductal gray (PAG) as the bladder expands, and the level of afferent nerve firing is proportional to bladder volume. The PAG relays this information to the cerebral cortex in humans, allowing for conscious input over the voiding reflex (Noack, 2012). Higher mammals, such as other primates, cats, and dogs, share this conscious control, though mechanisms differ in rodent models. Control of micturition is predominantly mediated by the pontine micturition center, also known as Barrington's nucleus (Bar), in the brainstem. Bar neurons project to the sacral spinal cord, with efferent projections to the detrusor and sphincter. Conscious control over the external urethral sphincter (EUS) is a learned behavior. The decision to relax the sphincter at sub-threshold pressures or the decision to maintain sphincter tonus at threshold pressures overrides the voiding reflex in potty-trained children and adults; in older adults with cognitive dysfunction, a proposed cause of incontinence episodes includes loss of EUS control and a degeneration of control over the voiding reflex.

## Aging Is Associated With Decreased Precision of Control

**FIGURE 1:**
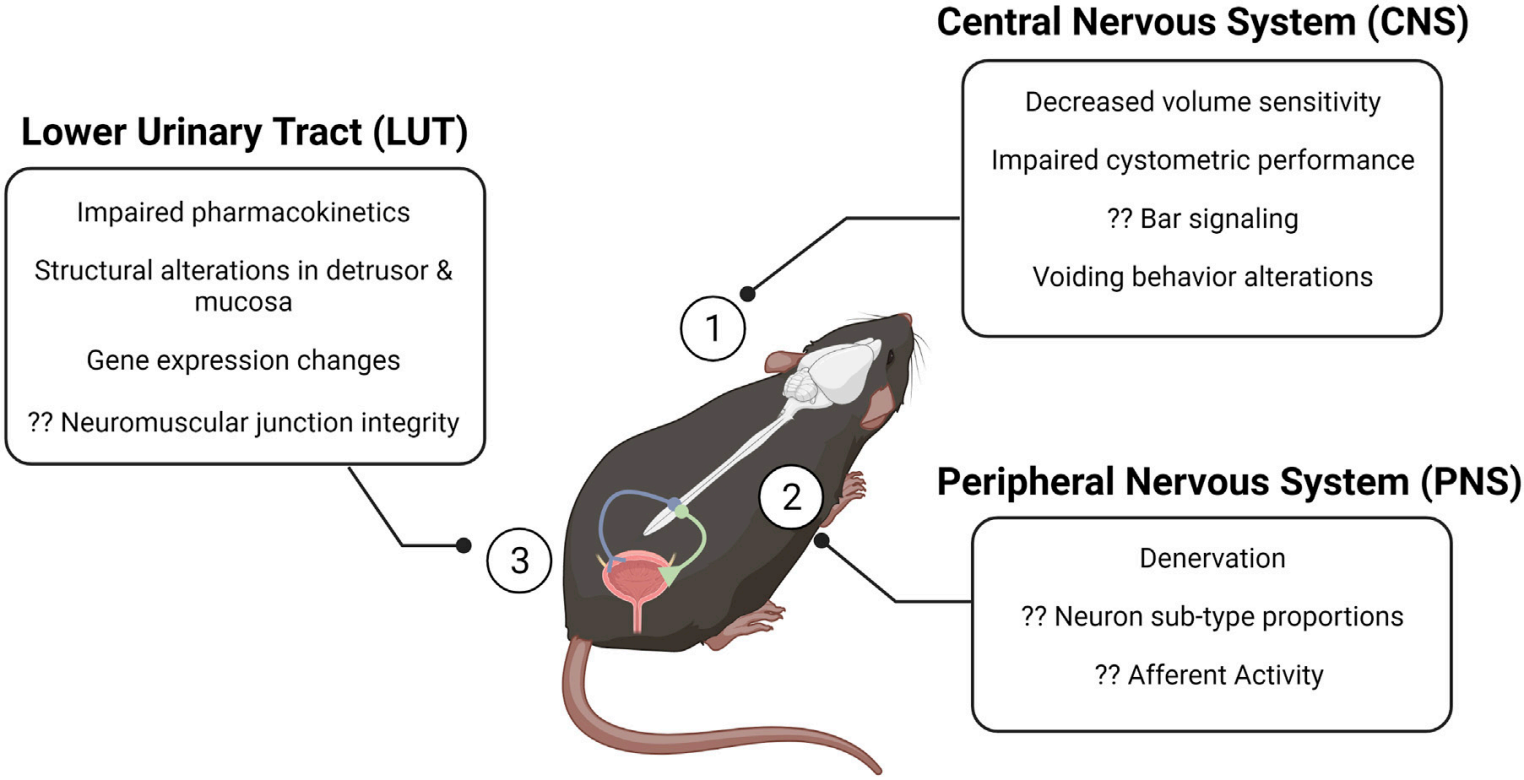
The aging brain–bladder axis of the mouse at a glance. The brain–bladder axis consists of the CNS (1), the PNS (2), and the LUT (3). The alterations described in the call-out boxes summarize gross changes in each region that have been associated with aging. Question marks denote gaps in the field. Created with BioRender. CNS, central nervous system; PNS, peripheral nervous system; LUT, lower urinary tract.

Functional changes in the urinary bladder are common in older adults, regardless of if they are symptomatic. The phenomena of overactive bladder (OAB) and underactive bladder (UAB) describe symptom complexes; diagnosis of detrusor underactivity (DU) or detrusor overactivity (DO) require clinical urodynamic assessment (Haylen et al., 2010). DU and DO can occur in asymptomatic individuals, through criteria assessing if there are insufficient or inappropriate detrusor contractility during supraphysiologic filling for DU and DO, respectively. The study of these conditions has revolutionized through cystometric evaluation—a comparable technique to urodynamics for the assessment of bladder function in animal models—including the development of the mouse cystometric model in 2010 (Fraser et al., 2020; Smith and Kuchel, 2010). Bladder diaries have been useful in the non-invasive assessment of frequency and urine output in humans; metabolic cages and voiding spot assays have proven beneficial in obtaining similar metrics in intact rodent models while permitting the study of the effects of aging and/or treatments over time (Figure 1). Though CNS control over micturition differs between rodents and humans, the overall effect is shared: aging increases the likelihood of incomplete bladder emptying and decreased sensitivity to volume (Triguero et al., 2014). Afferent data relaying bladder volume to the CNS provides the primary input from which the Bar integrates: alterations in firing rates, fiber properties, and populations of afferent fibers recruited can impede the ability of the CNS to effectively relay commands appropriate for a given volume. There is an inverse relationship between age and afferent firing rate; aged systems are, in general, less reliable in transmitting accurate information on bladder content.

Like many other faculties, the CNS suffers in the aged environment. Functional MRI (FMRI) studies have demonstrated decreased blood flow, brain shrinkage, and metabolic dysfunction in the brains of older adults (Smith et al., 2015; Andersson et al., 2017). In older adults, brain tissue loss, protein dysregulation, and degeneration/demyelination directly contribute to LUTS. Further evidence can be found in neurodegenerative conditions: multiple sclerosis (Ramasamy and Smith, 2021), Alzheimer's disease (Lee et al., 2014), and Parkinson's disease (Campeau et al., 2011) all have associated LUTS.

## Age-Associated Structural and Functional Alterations of the Bladder

During urine storage, adrenergic (sympathetic) inputs from the hypogastric nerve act on beta-adrenoceptors in the bladder wall, inducing relaxation, and act on alpha-adrenoceptors in the urethra, inducing contraction. Together, this allows for accommodation of increasing bladder volumes while maintaining relatively low pressure. Adrenergic and cholinergic receptor expression decreases with aging at both the mRNA and protein levels (Yoshida et al., 2001; Hardy et al., 2019). Emptying of the bladder is mediated through cholinergic (parasympathetic) activation of muscarinic receptors in the detrusor; parasympathetic outflow to the bladder simultaneously inhibits sympathetic and pudendal (somatic) outflow to the EUS, allowing for simultaneous contraction of the detrusor and relaxation of the EUS. Notably, EUS tonus has been observed to decrease with aging (Pfisterer et al., 2006). The deficits in detrusor contractility are thought to be due to inappropriate recruitment of collagen fibers, leading to increased stiffness (Cheng et al., 2018). Expulsive strength is persevered with aging, alluding to control—not tissue integrity—as the likely culprit of detrusor deficits (Mitteldorf, 2010; Smith et al., 2012). Both adrenergic and muscarinic responses are negatively impacted by aging, leading to decreased precision of neural control in the periphery (Lluel et al., 2000). In addition to neuronal signals, the detrusor is also under the control of paracrine factors, released from the urothelium in response to distension (Birder and Andersson, 2013). Excitatory molecules, such as ATP, and inhibitory molecules, such as nitric oxide (NO), are released locally, acting on detrusor myocytes and afferent fibers in the lamina propria. The release of stretch-induced and stimulation-induced ATP decreased with aging (Nishikawa et al., 2020). Age-associated deficits in cell morphology, mitochondria dysfunction, and increased production of reactive oxygen species have also been reported (Birder, 2020); the disruptions of post-synaptic structure and function are also implicated in neural control dysfunction.

## Discussion

LUTS are a common, costly problem: billions of dollars are spent annually on the treatment and management of incontinence and related urinary problems. Nearly half of women over 65 years experience urinary incontinence—allowing for acceptance of LUTS as “the new normal” with aging—yet many remain continent throughout their lives, implying simply possessing an old bladder is not the sole prerequisite for developing urinary dysfunction and/or symptoms. LUTS significantly decrease quality of life and are a driving force in institutionalization. Available pharmacologic therapeutics pose many unfortunate and undesirable side effects: anticholinergic medications, for example, have been associated with increased cognitive symptoms with little improvement in LUTS. Surgical interventions can be challenging in older adults, limiting the realistic use of neuromodulatory devices for sacral nerve stimulation.

Aging negatively impacts function across many systems, including the CNS. Vascular deficits are observed with aging: ischemia in the brain and bladder have been demonstrated, undoubtedly affecting urinary function (Andersson et al., 2017). Alterations in the biomechanics of the bladder provide mechanistic insight into compliance changes: old bladders stiffen at lower pressures than young bladders. This in turn prevents adequate distension—a necessary requirement for an effective afferent data stream—posing a potential mechanism for loss of volume sensitivity with aging. The side effects of traditional pharmacologic treatment of LUTS complicate their use in the geriatric patient (Risacher et al., 2016; Green et al., 2017); comorbidities often exist with LUTS, thus complicating surgical avenues as well (Katz et al., 2019). Investigations of urotoxic and uroprotective factors have shown support for the geroscience hypothesis: the balance between these compounds pose a clear target for therapeutic intervention, with promising results in animal models: the PNPase inhibitor 8-AG acts to reduce urotoxic by-products while increasing uroprotective factors (Birder et al., 2020). Treatment with 8-AG conferred structural alterations as well, rejuvenating structural morphology to that more reminiscent of the young animals (Birder et al., 2021). How interventions such as this impact neuronal signaling remains to be explored. Many gaps in the field remain—the identification of denervated neurons, signaling changes in the dorsal root ganglion (DRG), and alterations in neuromuscular junction integrity have not been thoroughly explored regarding neural control of micturition. Future investigations incorporating paracrine influence from the urothelium on neuronal transmission and release will further advance the field.
